# ITEP: An integrated toolkit for exploration of microbial pan-genomes

**DOI:** 10.1186/1471-2164-15-8

**Published:** 2014-01-03

**Authors:** Matthew N Benedict, James R Henriksen, William W Metcalf, Rachel J Whitaker, Nathan D Price

**Affiliations:** 1 Department of Chemical and Biomolecular Engineering, University of Illinois at Urbana-Champaign, 600 S. Mathews Ave., Urbana, IL 61801, USA; 2 Department of Microbiology, University of Illinois at Urbana-Champaign, 601 S. Goodwin Ave., Urbana, IL 61801, USA; 3 Institute for Genomic Biology, University of Illinois at Urbana-Champaign, 1206 W. Gregory Dr., Urbana, IL 61801, USA; 4 Institute for Systems Biology, 401 Terry Ave. N., Seattle, WA 98109, USA

**Keywords:** Comparative genomics, Clustering, Curation, Database, Metabolic networks, Orthologs, Pan-genome, Phylogenetics

## Abstract

**Background:**

Comparative genomics is a powerful approach for studying variation in physiological traits as well as the evolution and ecology of microorganisms. Recent technological advances have enabled sequencing large numbers of related genomes in a single project, requiring computational tools for their integrated analysis. In particular, accurate annotations and identification of gene presence and absence are critical for understanding and modeling the cellular physiology of newly sequenced genomes. Although many tools are available to compare the gene contents of related genomes, new tools are necessary to enable close examination and curation of protein families from large numbers of closely related organisms, to integrate curation with the analysis of gain and loss, and to generate metabolic networks linking the annotations to observed phenotypes.

**Results:**

We have developed ITEP, an Integrated Toolkit for Exploration of microbial Pan-genomes, to curate protein families, compute similarities to externally-defined domains, analyze gene gain and loss, and generate draft metabolic networks from one or more curated reference network reconstructions in groups of related microbial species among which the combination of core and variable genes constitute the their "pan-genomes". The ITEP toolkit consists of: (1) a series of modular command-line scripts for identification, comparison, curation, and analysis of protein families and their distribution across many genomes; (2) a set of Python libraries for programmatic access to the same data; and (3) pre-packaged scripts to perform common analysis workflows on a collection of genomes. ITEP’s capabilities include *de novo* protein family prediction, ortholog detection, analysis of functional domains, identification of core and variable genes and gene regions, sequence alignments and tree generation, annotation curation, and the integration of cross-genome analysis and metabolic networks for study of metabolic network evolution.

**Conclusions:**

ITEP is a powerful, flexible toolkit for generation and curation of protein families. ITEP's modular design allows for straightforward extension as analysis methods and tools evolve. By integrating comparative genomics with the development of draft metabolic networks, ITEP harnesses the power of comparative genomics to build confidence in links between genotype and phenotype and helps disambiguate gene annotations when they are evaluated in both evolutionary and metabolic network contexts.

## Background

Technological advances in DNA sequencing have led to rapid increases in sequencing throughput and a decrease in sequencing cost [1]. These advances have enabled comparative studies of the whole genomes of many related species [2]. Such genome analyses have provided valuable insights into evolutionary mechanisms, diversity, and adaptability of life to environmental variation [3-5] as well as key trait variations among industrially or medically important strains [6-9].

Identifying orthologs and orthologous protein families is an important step towards understanding and interpreting genome variation [10]. However, there is no single method that correctly predicts orthology in all cases, leading to the development of many different methods targeting different applications [11]. Due to the use of different algorithms and parameters used to perform clustering, automatically computed databases of orthologs often predict different protein families for the same proteins [12,13]. Since orthologs are often taken to have the same function, these differences lead to differences and thus to uncertainty in the predicted functions of the genes [11].

Further confounding the ability to automatically infer protein function, clustering efficacy depends on the evolution rate of those families, which can vary widely [14]. The need to carefully curate protein functions and gene calls is also compounded by a rapid increase in the number of incomplete genomes [15], including the approximations to single-species genomes that arise from metagenomic assemblies [16]. Careful examination of gene calls and functional annotations is particularly important for accurately assessing the gain and loss of function in these incomplete genomes because genes are often left uncalled or incorrectly annotated due gene fragmentation or sequencing errors (leading to erroneous frame shifts or nonsense mutations).

A number of software packages have been developed to integrate orthologous group identification, visualization tools, and common comparative analyses based on protein content [17-24]. However, due to the challenges cited above, many of these analyses require manual curation, which is difficult to scale to hundreds of genomes. Additional tools are necessary to help researchers curate annotations and evaluate the integrity of protein families across related genomes.

We present ITEP, a modular bioinformatics toolkit for the generation, curation, and analysis of protein families across closely-related microbial genomes in which the combination of core and variable genes constitute their "pan-genomes". The toolkit provides a consistent command-line interface between a user’s genomic data and existing tools for protein family prediction by clustering, ortholog detection, analysis of functional domains, identification of core and variable genes and gene regions, alignments and trees, cluster curation, and the integration of cross-genome analysis and the generation of draft metabolic networks for study of metabolic network evolution. The toolkit makes it easier to identify and fix problems such as inaccurate annotations and missing (un-called) genes and to study the evolutionary history and physiological implications of the curated families. ITEP’s architecture enables researchers to rapidly develop their own customized comparative analysis workflows, which are easily automated, allowing users to focus their curation effort, rapidly generate and test hypotheses, and build accurate metabolic networks.

## Implementation

**Figure 1:**
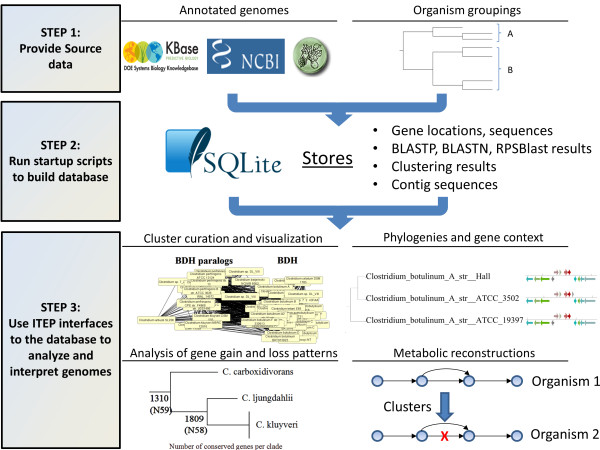
**Overview of the ITEP toolkit.** The ITEP toolkit is organized so that analyses can be performed in a three-step process. Step 1: The ITEP toolkit takes three inputs: Genbank files of genomes; user-defined groupings of input organisms in which to identify protein families; and clustering parameters that define the details of the clustering method used to identify the families. Step 2: The user calls provided setup scripts to build a SQLite database containing pre-computed data such as homology and clustering results. Step 3: After building the database, a user can use the provided interfaces to the database to identify core and variable genes, build protein and organism phylogenies, curate amd visualize protein families, or build draft metabolic reconstructions from a reference network. To accomplish ITEP interfaces with the SQLite database and many previously existing bioinformatics and programming packages [28-37].

The ITEP toolkit is a collection of Python and BASH scripts that interface with an SQLite database backend (Additional file 1) and a large number of existing tools to organize and analyze genomic content across related genomes (see Figure 1 and Additional file 2 for overview). The toolkit runs on Linux natively; a virtual machine is also provided that includes a complete ITEP installation, which can be run on any operating system (linked to from the project homepage at https://price.systemsbiology.net/itep). The toolkit includes: (1) convenient functions for genome importing and formatting, (2) modular analysis scripts that can be linked by piping to quickly and flexibly create workflows, (3) several convenient wrapper scripts that link other functions together to perform common analysis and visualization, and (4) a set of underlying Python libraries for programmatic data access. Interfaces are available for processing genomic data from the GenBank database [25], RAST [26], or the DOE KnowledgeBase [27]. Standard GenBank files (.gbk) from any other source may also be imported into ITEP by running them through a provided pre-processing script.

ITEP’s SQLite database stores information on gene locations, annotations and sequences, sequence homology data, *de novo*-computed protein families, protein similarities to externally defined orthologous groups (such as COGs), and the DNA sequence of each contig for every imported genome. Protein families are generated by creating a graph of similarities between proteins and running a clustering program (the most strongly supported clustering program is MCL [38], but a user can use any other clustering program as long as outputs are provided in the correct format). Setup scripts are provided to readily import this data into the SQLite database.

After the database is built, the user can use provided command-line scripts to access subsets of the data within it and perform the supported analyses (Figure 1). Most of the command-line access scripts are pipe commands, in which the output from one command is used as an input to another using pipes (|). This architecture allows users to rapidly prototype analyses and subsequently automate them in a Bash script. Many of the database access scripts generate tab-delimited outputs that are convenient for further command-line processing or import into spreadsheets. ITEP also contains commands to visualize phylogenies and gene context for genes in the database using freely available Python packages [28,29] or export data to standard formats such as FASTA alignments and Newick files which are widely supported in other visualization and bioinformatics software. Many of the same analyses implemented in the command-line scripts are also accessible programmatically via a set of Python libraries to aid developers who wish to build their own tools upon ITEP’s data structures. Finally, pre-packaged workflow scripts are provided for common analysis tasks such as the generation of concatenated core gene trees. These can be used to quickly obtain a result or as a working starting point from which to develop new analysis pipelines.

### *De novo* clustering for computation of protein families

Running the BLASTP program [30] all vs. all provides a graph of similarities between pairs of proteins, in which the genes are nodes and each significant pairing is an edge weighted by some similarity metric. The ITEP toolkit's setup scripts directly support the generation of protein families *de novo* by clustering these graphs using the Markov Cluster (MCL) algorithm [31]. The toolkit allows many different definitions of the homology graph: it can be generated from arbitrary subsets of organisms in the database with arbitrary cutoffs and inflation parameters (clustering sensitivity), and three scoring metrics that emphasize different aspects of the protein pair homology (Additional file 3) [31,39,40].

A user can also import results from any other orthologous family prediction method, allowing flexibility that is necessary due to differences in the strengths and weaknesses of individual algorithms. All downstream analyses (e.g. phylogenetic analysis of gene gain and loss) can then be performed in the same manner as if the clusters were generated using MCL. For example, a wrapper function is provided to interface between the ITEP database and OrthoMCL, a program that applies a percent identity cutoff between pairs of homologous proteins, identifies likely orthologs by using a modified bidirectional-best-hits approach, and then runs MCL to cluster the smaller subset of nodes and edges into protein families [32]. It thus performs MCL only on filtered subsets of the homologous pairs of organisms rather than simply applying a simple cutoff for a homology score. The consistent storage of clustering results from multiple different clustering methods in a single database enables users to easily compare the effects of the choice of clustering algorithm and the choices of organisms to cluster on the predicted protein families.

### Protein family curation and visualization tools

Several biological and non-biological variables can cause automatically computed protein families to be incorrect or incomplete, such as the presence of gene fusions or multiple-domain proteins, incomplete or inaccurate gene calling, sequence and/or functional divergence, and the lack of rate homogeneity in evolution rates. In light of these challenges and in order to increase confidence that conclusions about the evolution of protein families are correct, we have implemented tools to generate and visualize multiple alignments and trees for protein families, to study gene neighborhoods of genes in a family, to search for possibly missing genes, and to assess the function of proteins in the light of their conserved domain architecture.

**Figure 2:**
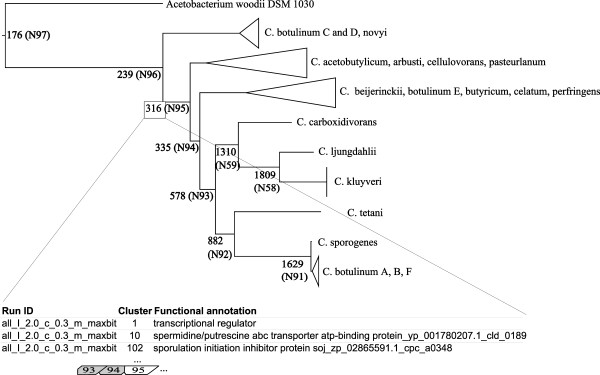
**Illustration of ITEP’s capabilities for studying gene gain and loss patterns across a phylogeny.** The node labels are the number of gene families (as computed by an MCL clustering of BLASTP results for both complete and draft genomes) that have at least one representative in each child of that node. Labels also contain a node identifier (N95) that can be used to look up the identities of all of the conserved families in tables outputted by the program. Examples of conserved families at node N95 are shown beneath the tree. The tree was generated from a concatenated alignment of ribosomal proteins uniquely identified in all of the genomes (17 families) with ITEP’s scripts, using FastTree [36] and a WAG model of evolution. Clusters were generated with the parameters: MCL clustering, inflation parameter of 2.0 (default for MCL), maxbit score, cutoff of 0.3. The tree was drawn with FigTree [43].

**Figure 3:**
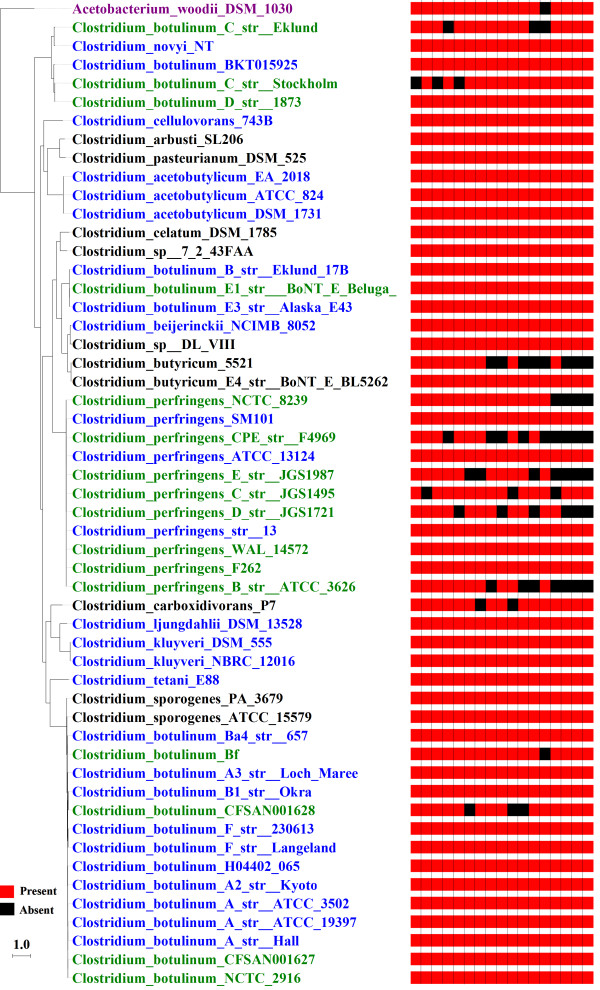
**Ribosomal proteins apparently missing in draft genomes and present in all complete genomes.** The heat map shows the presence (red) and absence (black) of the 17 ribosomal proteins that, according to RefSeq gene calls and the MCL clustering approach, were present in all complete Group 1 *Clostridia* genomes but missing in at least one draft genome within the same phylogenetic clades as the completely sequenced genomes. Blue strains: Completely sequenced genomes; green strains: draft genomes in the same clade as completely sequenced genomes; black strains: draft genomes in different clades from completely sequenced genomes. The tree is the same as that generated in Figure 2 and was visualized with ITEP scripts with some formatting changes (genome colors and column labels).

Multiple alignments and phylogenetic trees are useful to analyze the phylogenetic history of particular protein families and to sort out the potential presence of paralogs [41]. The ITEP toolkit contains convenient interfaces for generating protein and nucleotide alignments [33,34], curating alignments [35], and generating maximum-likelihood phylogenies [36,37]. ITEP’s tree visualization capabilities provide an interface between a user’s genomic data and the ETE Python package for tree manipulation and rendering [28]. The ITEP scripts include the option of appending gene neighborhood information to a protein tree, which is useful for identifying the functions of novel genes [23,42]. The user also has the option to attach numeric data (as a heatmap) or arbitrary text tables to any tree (see Figures 2 and 3).

To help identify missing genes, we have implemented an interface that links genomic data in ITEP to tBLASTn [44], which is useful for finding genes that are fragmented, miscalled (e.g. with frameshifts or nonsense mutations resulting from sequencing errors), or that are not yet annotated. The ITEP interface to tBLASTn identifies significant hits from a set of query genes to a particular genome (or set of genomes) in the database, and then automatically identifies whether the hit was to a called gene and whether the called gene was on the same strand as the hit. From this result, a researcher can examine and (if appropriate) add missing proteins to protein families. The gene neighborhood and tree generation and visualization scripts support the visualization of tBLASTn hits in their genetic context in the same manner as called genes (see Figure 3). We have also provided a tool that attempts to identify frame shifts, insertions, and nonsense mutation events from the tBLASTn results, which helps identify specific mutations that could lead to loss of function or that could indicate errors in the genome sequence.

**Figure 4:**
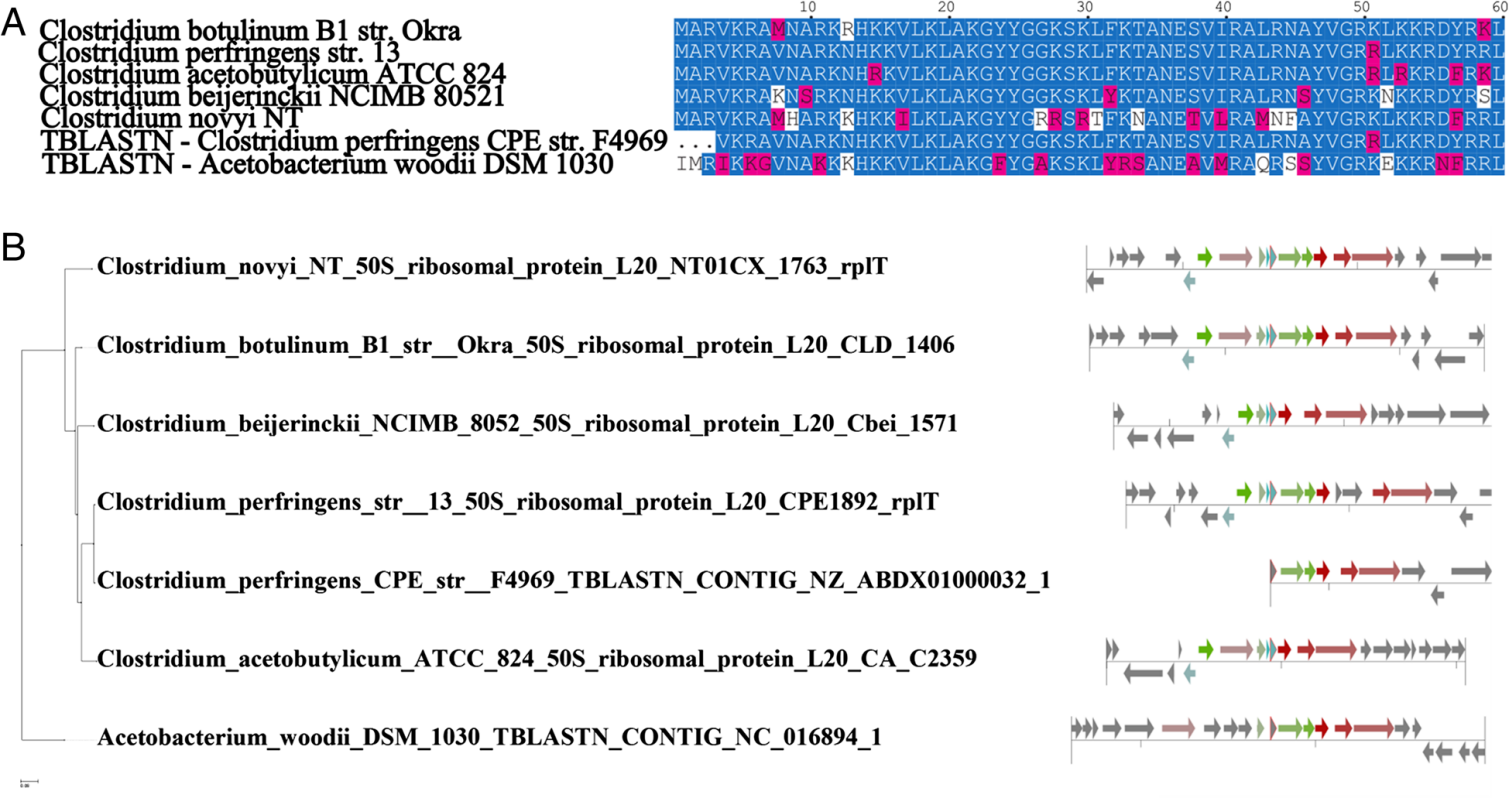
**Protein family curation with ITEP. (A)** A portion of the multiple alignment for the uncalled ribosomal protein L20 homologs in *Acetobacterium woodii* and *C. perfringens* str. CPE F4969, along with selected representatives of this protein from other *Clostridia*. Blue amino acids were conserved in more than 50% of the aligned proteins and pink amino acids are similar to the conserved acids. The figure in part (A) was generated by importing a multiple alignment generated by an ITEP script into the STRAP aligner [46]. **(B)** Gene neighborhoods for the proteins from part (A) attached to the maximum-likelihood phylogeny of the same proteins. Same-colored arrows indicate that the genes belonged to the same family according to MCL with the same parameters used to construct Figure 2. The visualization was done with an ITEP script.

Finally, to assist the curation of annotations, we have implemented automatic generation and storage of RPSBLAST hits to the NCBI CDD database [45]. The interface allows a user to rapidly search for the IDs of conserved domains that correspond to certain keywords (such as “purine synthesis”) and to identify all proteins in a genome that have significant homology to a specific set of conserved domains. ITEP also includes tools for identifying and visualizing all conserved domains that are found in a specific query protein or set of proteins (Figure 4), providing insight into the functions of those proteins.

### Analysis of core and variable gene content

Studying gene gain and loss and examining the core (conserved) and variable (non-conserved) genes in a collection of organisms can provide insights into the plasticity of cellular functions and can be used to identify genes that define a clade [47]. To assist such analyses, ITEP includes functions that identify interesting subsets of genes based on presence and absence patterns, such as genes that are present in *all* of a particular group of organisms (conserved genes), *any* members of a group (present genes), *only* members of that group relative to those all of the organisms to generate the protein families (unique genes), or *none* of the members of that group. The script can also optionally identify genes that are conserved in any given fraction of a group of organisms, allowing for some flexibility due to missed gene calls or divergent sequences. Finally, if an organism phylogeny is available (or built with other ITEP scripts), a tool is also available to identify presence and absence patterns based on each phylogenetic clade, allowing a researcher to, for example, identify all of the genes that are conserved in or unique to each individual species or all genomes in a clade.

### Integration with metabolic networks

A key reason to identify protein families is to use the results to propagate annotations and subsequently identify the physiological capabilities of an organism based on those of its relatives. In the context of genome-scale metabolic modeling, the predicted presence or absence of particular protein families may be used as evidence for the presence or absence of reactions in a metabolic network. In a metabolic network reconstruction, the relationship between a gene and the reactions catalyzed by the encoded enzyme is typically encoded in a Boolean gene-protein-reaction relationship (GPR), in which complexes and other sets of genes that must all be present for a reaction to occur are given an AND relationship, while isozymes or sets of genes with unknown relationships are given an OR relationship [48]. To assess whether a reaction is catalyzed or not within a cell, each associated gene is assigned a 1 (TRUE) if it is present and a 0 (FALSE) if it is absent, and then the GPR is logically evaluated. If the GPR evaluates to TRUE then the reaction is present and otherwise it is absent.

We have implemented a function in ITEP that directly evaluates Boolean gene-protein-reaction relationships associated with existing metabolic reconstructions of strains in the database based on the presence-absence calls of *de novo* clustering with arbitrary parameters. In this way, a researcher can rapidly generate draft metabolic network reconstructions based on genomic comparisons with one or more reference networks. Subsequently, these network reconstructions can be curated to generate high-quality models of each related organism.

## Results and Discussion

### Test data set

We chose to use the Group 1 Clostridia as a test case to illustrate capabilities of the ITEP toolkit. This metabolically diverse phylogenetic clade includes industrially important organisms such as the solventogenic organisms *Clostridium acetobutylicum* and *C. beijerinckii*, as well as several medically important strains such as *C. perfringes* and *C. botulinum*[49]. *C. botulinum* and *C. perfringes* genomes have both been heavily sampled, therefore providing the opportunity to study genetic differences at both species and at the genus-scale. In addition, manually-curated metabolic models are available for *C. acetobutylicum* ATCC 824 [50,51] and *C. beijerinckii* NCIMB 8052 [52], affording an opportunity to use ITEP to examine metabolic differences between these and the other *Clostridium* species in the clade.

The species belonging to the Group 1 Clostridia were determined based on the PATRIC database [53] and the ARB Living Tree 16S rRNA tree [54]. All complete and draft genomes from this group were downloaded from RefSeq in March 2013 (including plasmids) along with the genome of an outgroup organism, *Acetobacterium woodii*. Overall, 26 complete and 26 incomplete Clostridia genomes were downloaded and analyzed (see Additional file 3 for complete strain names and RefSeq accession numbers).

The test dataset was chosen to be relatively small for purposes of illustration. ITEP currently supports creation of databases containing up to about 200 genomes on a modern workstation with 1 TB of hard drive space, 16 GB of RAM, and 12 processors (using which all vs. all BLAST, MCL, and RPSBlast would take about 6 days altogether). Disk space and time requirements grow as O(N^2^) where N is the number of genomes.

In this example, MCL was used to perform clustering and predict protein families. The relative strengths of this and other methods for predicting protein function have been reviewed at length [13,55-57]. Importantly, if the user desires to use different algorithms for clustering, ITEP supports exporting subsets of BLAST data in formats convenient for import into clustering tools, importing the clustering results back into the SQLite database, and applying the same workflows as described here to interpret and curate them.

Complete tutorials for performing the analyses described in this section and many others are available in the package documentation (included as Additional file 4, matching the version of ITEP code provided as Additional file 5). A link to an up-to-date web version of this documentation and code is linked to from the project website (https://price.systemsbiology.net/itep).

### Analysis of gene gain and loss patterns across phylogeny

As a starting point for the analysis of the Group 1 *Clostridia* pan-genome, we used ITEP to compute the number of conserved gene families (one member or more in every organism) in each clade in the Group 1 *Clostridia* and in *A. woodii* (Figure 2). The results indicate that a large number of genes are conserved between closely related strains (such as *C. sporogenes* and *C. botulinum* A, B and F subtypes) but the number of conserved genes drops off rapidly as more diverse strains are added. The identities of the conserved genes can easily be extracted from ITEP and used to examine physiological differences between the clades of organisms and at what point a particular function was lost. In the same manner, ITEP can be used to identify gene families unique to each clade or those that are found in exactly one copy in each member. Importantly, the curation tools in ITEP can be used to verify conclusions drawn from analyzing these gain and loss patterns (see later sections for some examples).

### Comparison of draft and complete genomes and curation of protein families

Draft genomes are prevalent in many environmental studies, but because they are incomplete, presence and especially absence calls are inherently less certain for them than they are for complete genomes. The grouping capabilities of ITEP are useful for evaluating the quality of draft genomes by comparing their gene content with closely related closed genomes. To illustrate this, we have generated MCL clusters including two different groups of organisms with identical clustering parameters: one group contained only the completely sequenced Group 1 Clostridia species (blue genomes in Figure 3), while the other contained both the completely-sequenced genomes and the draft genomes for strains in the same phylogenetic clades as the completely-sequenced species (green genomes in Figure 3 - only those genomes in the same clade were used to minimize differences due to species divergence). By comparing the protein content in these two groups, we found that 561 protein families were conserved in all of the completely sequenced genomes, but that 270 of them (48%) were missing in at least one of the draft genomes in the same clades (see Additional file 3 for a complete list). The protein families that appeared to be missing in some of the draft Group 1 Clostridia genomes but not the complete ones covered many cellular subsystems, including 17 ribosomal protein families (Figure 3) and other widely conserved proteins such as the cell division protein FtsZ.

When a highly conserved gene appears to be absent in a particular genome but does not have a congruent loss pattern on the phylogenetic tree, these are candidates for missing or wrong annotations or gene calls. Importantly, ITEP includes ways to search for apparently missing genes in the incomplete genomes, making it possible to identify and correct certain types of gene calling and annotation errors. As an example, we have used the tBLASTn wrapper script in ITEP to search for copies of the L20 ribosomal protein in all of the Group 1 Clostridia and in *Acetobacterium woodii*. The search revealed a complete, uncalled copy of the L20 protein in *A. woodii* and an uncalled fragment (on the end of a contig) of a L20 protein in *C. perfringens* CPE F4969.

To find evidence that these were real L20 proteins, we used ITEP scripts to pull the homologous sequences suggested by tBLASTn out of the database, align them, and build a maximum-likelihood tree containing these proteins with neighborhoods mapped onto the tree. The multiple alignment confirmed that the newly identified L20 homologs are very similar to called ribosomal proteins in closely-related complete genomes (Figure 4A and Additional file 6), while mapping the neighborhoods of the uncalled genes revealed significant conservation of gene neighborhoods (Figure 4B), supporting the hypothesis that the identified proteins are really L20 ribosomal proteins and should be included in the gene annotation. The same methodology can also be applied to search for apparently missing metabolic or regulatory genes, which would help fill in gaps that appear when generating models of cellular physiology. In this way, the challenge of accurate gene annotation can be approached both from the bottom up (gene orthology) and top down (relationship to physiological functions), tying together microbial phylogeny and physiology.

### Draft metabolic reconstruction and curation of metabolic protein families

The comparative analysis capabilities of ITEP can be used to generate draft metabolic networks as a starting point for generating high-quality metabolic models of organisms based on their similarity (or lack of similarity) to related genomes. To illustrate this capability, we have generated draft metabolic networks of each completely-sequenced Group 1 Clostridia strain using the published *C. beijerinckii* model [52] as a reference. This model was chosen as a reference because it is the most recent and most complete model of a member of the Group 1 *Clostridia* that has been published. We found that the presence and absence calls for metabolic functions in the other *Clostridia* were strongly dependent on the chosen homology cutoff: with a relatively stringent cutoff of 0.5, some organisms (such as *C. tetani*) appeared to be missing more than half of the 874 gene-associated metabolic reactions in the *C. beijerinckii* metabolic reconstruction, and even with a very lenient cutoff of 0.1, at least 100 of them were missing in each other organism (see Additional file 3). These missing reactions create gaps in the metabolic network that represent either real differences in physiology or incorrect absence calls due to methodological issues such as incorrect clustering, mis-annotation, or missing gene calls.

**Figure 5:**
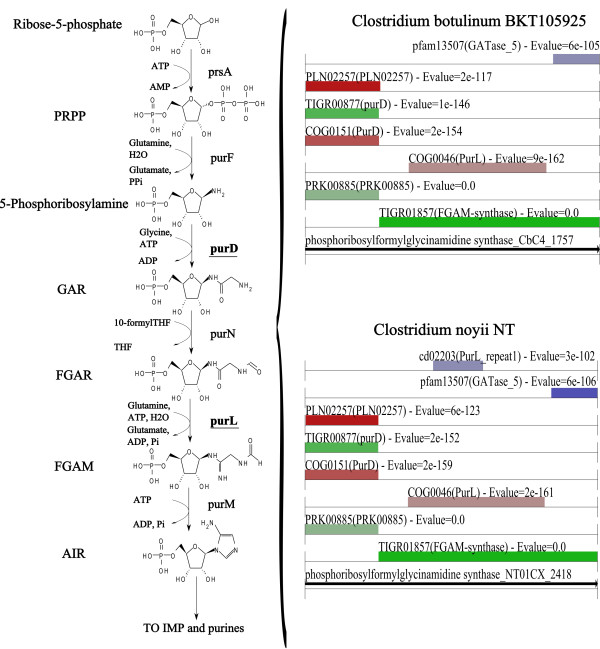
**Curation of a metabolic protein family by comparison with conserved domains.** Left side: a portion of the purine synthesis pathway in the group 1 Clostridia. Right side: conserved domain architecture of two *purD-purL* fusions in the group 1 Clostridia as computed and displayed by ITEP tools (with minor formatting changes). The comparison makes it clear that these two proteins are fusions of *purD* and *purL*. See list of abbreviations for full compound names. Only hits to conserved domains with E-values better than 1E-100 are shown.

The presence of gaps in reconstructed networks makes it difficult to turn them into functional metabolic models [58]. The comparative genomics capabilities of ITEP can be used to help identify genes that fix gaps in metabolic pathways (either those generated by using ITEP's clustering capabilities or those built using other tools). For example, the draft metabolic reconstructions for *Clostridium botulinum* BKT105925 and *C. novyi* NT based on MCL clustering were predicted to lack the *purD* enzyme necessary for purine synthesis (down to a homology cutoff of 0.1 maxbit score). No genes were annotated to perform this function in the source GenBank files for these genomes. In an attempt to fill this gap, we used ITEP to perform a tBLASTn search against these two organisms using the copy of *purD* from *C. beijerinckii* (Cbei_1060) as a reference. Interestingly, we found a very strong homology between the *C. beijerinckii purD* and the N-terminal end of much larger proteins in *C. botulinum* BKT105925 and *C. novyi* NT (CbC4_1757 and NT01CX_2418, respectively). Searching these genes against the RPSBLAST results that were stored in the ITEP database revealed that the large proteins from *C. botulinum* BKT105925 and *C. novyi* NT are in fact fusions of *purD* and *purL* (Figure 5), in agreement with the assignments based on MetaCyc [59], RAST [26], and the SEED [60]. Therefore, the gap in the metabolic network can be fixed by assigning the same function to both of these genes, making simulations performed using other tools [61-63] more accurate.

## Conclusions

The ITEP toolkit integrates a large number of existing bioinformatics tools into a single cohesive, flexible framework for comparative analysis of physiological variation in microbial pan-genomes. The modular design of the toolkit makes it straightforward to add additional functionality to the toolkit, as illustrated by our implementation of novel tools for generation of draft metabolic reconstructions from a curated reference network. It also makes the analysis very flexible, empowering researchers to quickly develop analysis workflows while also providing a wide array of tools for curation of annotations and gene calls. The ability to rapidly curate protein families and propagate metabolic networks from reference organisms to related strains will streamline the process of generating high-quality physiological and evolutionary hypotheses and ultimately lead to an improvement in the inter-genome consistency of metabolic models of microbes.

## Availability and requirements

**Project name:** ITEP: Integrated Toolkit for Exploration of microbial Pan-genomes.

**Project home page:**https://price.systemsbiology.net/itep

**Operating system(s):** Linux; A virtual machine is available that can be run on any platform supported by VirtualBox (link is on the project home page).

**Programming language:** Python (2.6 or 2.7), Bash

**Other requirements:** SQLite3+, MCL, NCBI BLAST+, Python modules: Biopython (1.61), Numpy, Ruffus, and ETE2; others are optional depending on usage.

**License:** GNU GPL2.0+

**Any restrictions to use by non-academics:** None

## Abbreviations

AIR: Aminoimidazole ribotide; BLAST: Basic local alignment search tool; FGAM: 5′-Phosphoribosylformylglycinamidine; FGAR: N-Formylglycinamide ribonucleotide; GAR: Glycinamide ribonucleotide; GPR: Gene-Protein-Reaction relationship; ITEP: Integrated toolkit for the exploration of pan-genomes; MCL: Markov cluster (clustering algorithm); PRPP: 5-Phosphoribosyl 1-pyrophosphate; RAST: Rapid annotation using subsystem technology; tBLASTn: Translated BLAST against nucleotides.

## Competing interests

The authors declare that they have no competing interests.

## Authors’ contributions

MB conceived the idea and implemented the toolkit. JH contributed code to the toolkit, participated in the toolkit design, and participated in the interpretation of the results. WM, RW and NP contributed ideas to improve the toolkit and participated in the interpretation of the results. All authors participated in the writing and revision of the manuscript. All authors read and approved the final manuscript.

## Supplementary Material

Additional file 1 Database schema for SQLite database. Click here for file

Additional file 2 ITEP architecture overview figure. Click here for file

Additional file 3 **List of genomes used in the analysis; List of protein families conserved in the complete group 1 Clostridia but not in the draft ones within the same clades; List of valid clustering metrics; list of presence and absence of metabolic reactions in the Group 1 Clostridia compared to *C. beijerinckii *** **as a function of cutoff.** Click here for file

Additional file 4 **Text version of the tutorials for initial release (ZIP).** The files are in Markdown format which can be viewed in many browsers using plugins such as the Markdown Viewer (https://addons.mozilla.org/en-US/firefox/addon/markdown-viewer/). Click here for file

Additional file 5 Source code for initial ITEP release. Click here for file

Additional file 6 **Complete PDF file for the Strap alignment in Figure** 4**A.** Click here for file
