# Supplementary figures and images for: ITEP: An integrated toolkit for exploration of microbial pan-genomes
Source: BMC Genomics. 2014 Jan 3;15:8. doi: 10.1186/1471-2164-15-8 (PMC3890548; doi:10.1186/1471-2164-15-8)

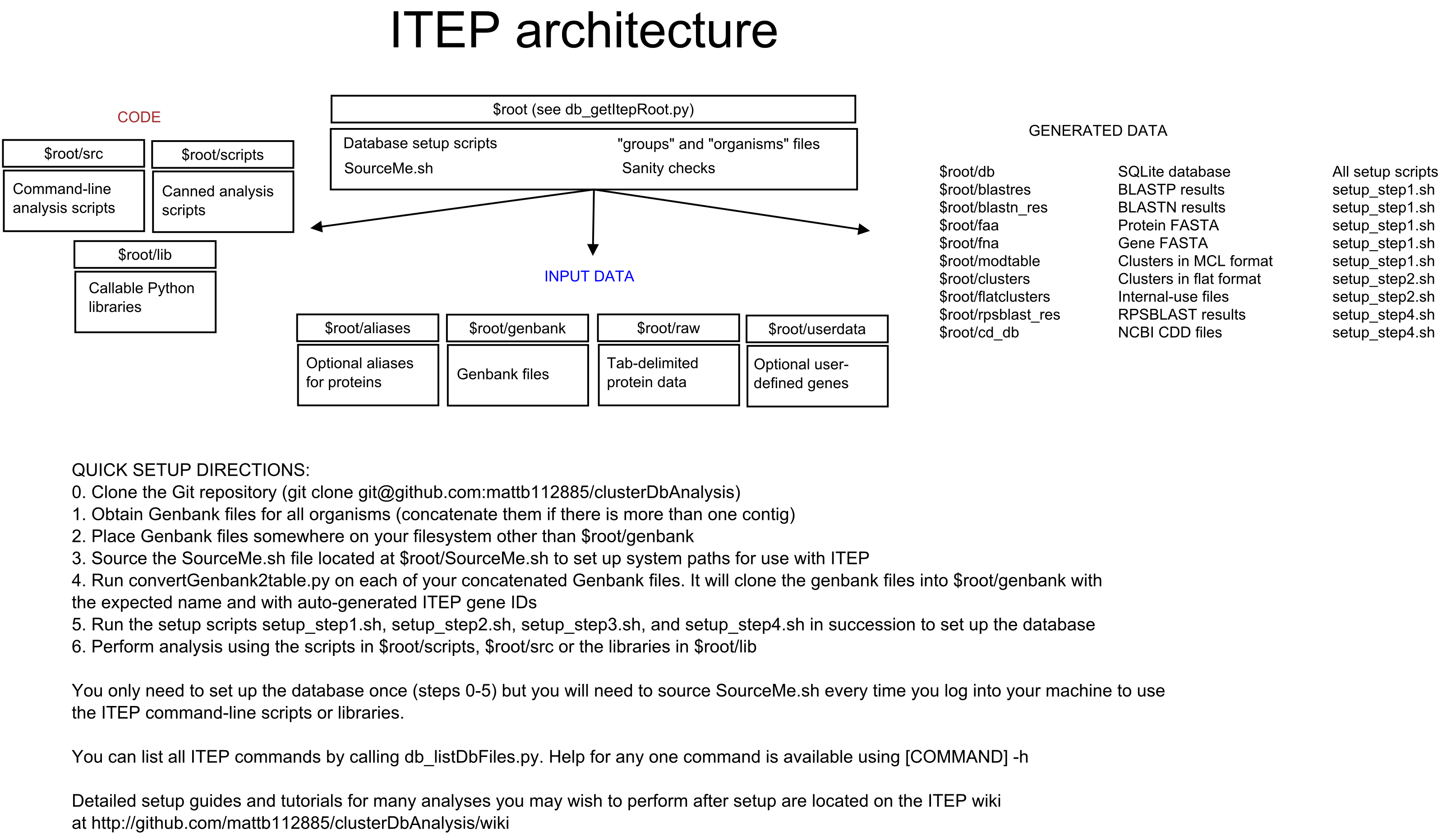

Supplement: Additional file 2 — ITEP architecture overview figure. [file 1471-2164-15-8-S2.png]

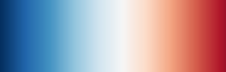

Supplement: Additional file 5 — Source code for initial ITEP release. [file 1471-2164-15-8-S5.zip › clusterDbAnalysis-master/src/internal/Colormap.png]

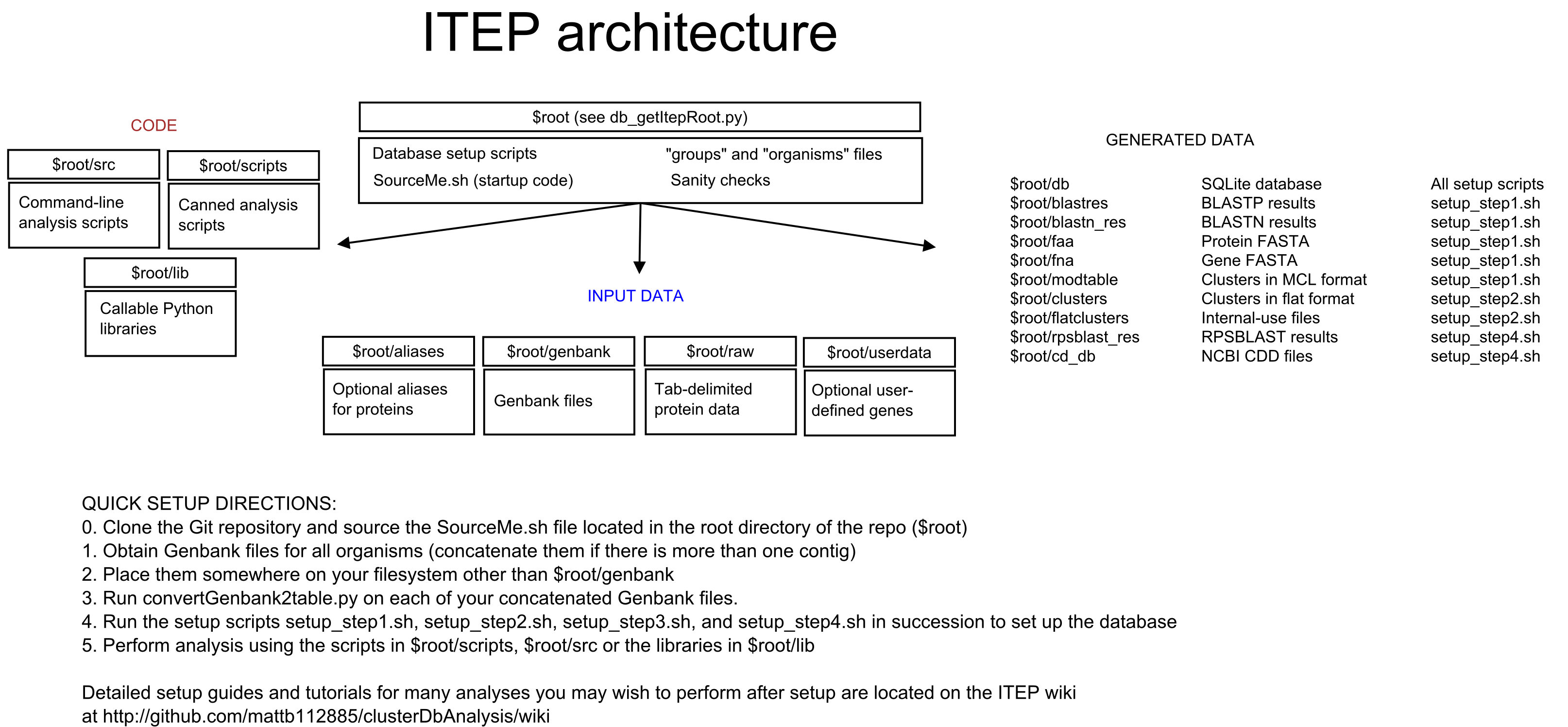

Supplement: Additional file 5 — Source code for initial ITEP release. [file 1471-2164-15-8-S5.zip › clusterDbAnalysis-master/src/internal/ITEP_overview_figure.png]
