# Supplementary material for: ITEP: An integrated toolkit for exploration of microbial pan-genomes
Source: BMC Genomics. 2014 Jan 3;15:8. doi: 10.1186/1471-2164-15-8 (PMC3890548; doi:10.1186/1471-2164-15-8)
Supplement: Additional file 6 — Complete PDF file for the Strap alignment in Figure 4A. [file 1471-2164-15-8-S6.pdf]

```
                                                             10        20        30        40        50        60        70        80
Clostridium_botulinum_B1_str__Okra                       1   MARVKRAMNARKRHKKVLKLAKGYYGGKSKLFKTANESVIRALRNAYVGRKLKKRDYRKLWIARINAATRMNGLSYSKFMNGIKN
Clostridium_perfringens_str__13                          1   MARVKRAVNARKNHKKVLKLAKGYYGGKSKLFKTANESVIRALRNAYVGRRLKKRDYRRLWIARINAATRMNGLSYSRFMNGMKL
Clostridium_acetobutylicum_ATCC_824                      1   MARVKRAVNARKNHRKVLKLAKGYYGGKSKLFKTANESVIRALRNAYVGRRLRKRDFRKLWIARINAATRINGLSYSKFINGIKL
Clostridium_beijerinckii_NCIMB_8052                      1   MARVKRAKNSRKNHKKVLKLAKGYYGGKSKLYKTANESVIRALRNSYVGRKNKKRDYRSLWIARINAATRINNLSYSKFMNGIKL
Clostridium_novyi_NT                                     1   MARVKRAMHARKKHKKILKLAKGYYGRRSRTFKNANETVLRAMNFAYVGRKLKKRDFRRLWIARINAAARMNGLSYSKFMNGIKL
TBLASTN_CONTIG_Clostridium_perfringens_CPE_str__F4969    1   ...VKRAVNARKNHKKVLKLAKGYYGGKSKLFKTANESVIRALRNAYVGRRLKKRDYRRLWIARINAATRMNGLSYSRFMNGMKL
TBLASTN_CONTIG_Acetobacterium_woodii_DSM_1030            1   IMRIKKGVNAKKKHKKVLKLAKGFYGAKSKLYRSANEAVMRAQRSSYVGRKEKKRNFRRLWITRINAGARMYDLSYSKFMFGLKQ

                                                                  90        100       110
Clostridium_botulinum_B1_str__Okra                      86   AGIDINRKMLSEIAINDPKAFAELVDVAKKQLNA
Clostridium_perfringens_str__13                         86   AGVDINRKMLSEIAINDPKAFADLVELAKKHLNA
Clostridium_acetobutylicum_ATCC_824                     86   AGIDMNRKMLSEIAINDPKAFSELVEVAKKQINA
Clostridium_beijerinckii_NCIMB_8052                     86   AGIDINRKMLSEIAINDPKAFTELVEVAKKQLNA
Clostridium_novyi_NT                                    86   AGINMNRKMLSEIAINDEKAFADLVEVAKKQLNA
TBLASTN_CONTIG_Clostridium_perfringens_CPE_str__F4969   83   AGVDINRKMLSEIAINDPKAFADLVELAKKHLNA
TBLASTN_CONTIG_Acetobacterium_woodii_DSM_1030           86   AGVEIDRKILADLAMNDINAFKDLVEVSKKNLN.
```

X non conserved

X similar

X ≥ 50% conserved
